# Influence of Codon Bias on Heterologous Production of Human Papillomavirus Type 16 Major Structural Protein L1 in Yeast

**DOI:** 10.1100/2012/979218

**Published:** 2012-05-02

**Authors:** Milda Norkiene, Alma Gedvilaite

**Affiliations:** Institute of Biotechnology, Vilnius University, Graiciuno 8, Vilnius, Lithuania

## Abstract

Heterologous gene expression is dependent on multistep processes involving regulation at the level of transcription, mRNA turnover, protein translation, and posttranslational modifications. Codon bias has a significant influence on protein yields. However, sometimes it is not clear which parameter causes observed differences in heterologous gene expression as codon adaptation typically optimizes many sequence properties at once. In the current study, we evaluated the influence of codon bias on heterologous production of human papillomavirus type 16 (HPV-16) major structural protein L1 in yeast by expressing five variants of codon-modified open reading frames (OFRs) encoding HPV-16 L1 protein. Our results showed that despite the high toleration of various codons used throughout the length of the sequence of heterologously expressed genes in transformed yeast, there was a significant positive correlation between the gene's expression level and the degree of its codon bias towards the favorable codon usage. The HPV-16 L1 protein expression in yeast can be optimized by adjusting codon composition towards the most preferred codon adaptation, and this effect most probably is dependent on the improved translational elongation.

## 1. Introduction

The production of functional proteins in heterologous hosts is an important issue of modern biotechnology. However, often it is difficult to generate recombinant proteins outside their original context. Protein expression is dependent of multistep processes involving regulation at the level of transcription, mRNA turnover, protein translation, and posttranslational modifications leading to the formation of a stable product [1]. Currently, it is accepted that codon bias has a crucial role in heterologous gene expression and that nonoptimal codon content can limit gene expression due to the shortage of available tRNAs in the heterologous host resulting in slowed elongation of the nascent peptide or premature termination of translation [2–5]. The observation that highly expressed genes have strong codon bias towards “preferred” codons is used to substitute the codons throughout the length of the target sequence into preferred high-frequency codons from the expression host. These changes improved the synthesis and yield of some heterologous proteins in different organisms [1, 5, 6].

Nonetheless, the codons that are adapted to the efficient elongation of endogenous genes may not always correspond to the efficient codons for heterologous genes, because overexpression often causes amino acid starvation what leads to changes and disbalance of charged tRNA pools [5]. It was shown that endogenous genes encoding amino acid biosynthetic enzymes that are essential during amino acid starvation preferentially use codons that are poorly adapted to the typical pool of charged tRNAs but are well adapted to starvation-induced tRNA pools [7, 8]. For this reason, the approach of codon bias optimization by adjusting codon usage to match cellular tRNA abundances in standard conditions now is changing to “codon harmonization” [9]. This new approach puts some nonpreferred codons in positions that correspond to predicted protein domain boundaries, and “codon sampling” adjusts the codon usage to reflect the overall usage in the target genome [10]. In the absence of tRNA abundance estimates, codon frequencies in the target genome are sometimes used.

Despite accumulating information about the impact of codon bias on the heterologous gene's expression, it is still unclear what approach for codon adaptation should be used designing putative transgene sequences as these approaches have not been systematically compared against each other. The codon adaptation typically optimizes many sequence properties at once, and in most cases, it is difficult to determine which parameter causes the observed differences in heterologous gene expression.

It was shown that papillomavirus late mRNAs may not be efficiently translated in undifferentiated cells due to a mismatch of codon usage and tRNA availability [11]. Optimization of human papillomavirus (HPV) L1 genes by introducing favorable human and plant codons improved production of L1 protein in human and plant cells [12, 13].

In the current study, we aimed to evaluate the influence of codon bias on heterologous generation of HPV type 16 (HPV-16) major structural protein L1 in yeast by expressing five different codon-modified open reading frames (OFRs) encoding HPV-16 L1 protein.

## 2. Materials and Methods

### 2.1. Generation of HPV-16 L1 Protein Expression Plasmids

All DNA manipulations were performed according to standard procedures [14]. Enzymes and kits for DNA manipulations were purchased from Termo Scientific Fermentas (Vilnius, Lithuania). The codons in the sequence of native HPV-16 isolate 114/K L1 gene [15] L1-Pv were either optimized according to the frequency analysis of codons determined (http://www.kazusa.or.jp/codon/) for overall yeast *S. cerevisiae* proteins (L1-Sc), plant proteins (i.e., *Solanum tuberosum* L1-Pl, EMBL accession no. AJ313181), and mammalian proteins (*Homo sapiens* L1-Hm, accession no. AJ313179) or adapted for expression in *E. coli* (L1-Ec) allowing some deviations from the strictly optimized codon usage. The genes encoding L1-Sc and L1-Ec of the HPV-16 were synthesized in GenScript (Piscataway, NJ, USA). The genes encoding L1-Pv, L1-Pl, and L1-Hm [12] were kindly provided by Martin Müller (German Cancer Research Center). 

The L1-encoding ORFs were cloned into the yeast vector pFX7 [16], allowing the selection of yeast transformants by permitting resistance to formaldehyde [17]. The sequences of the inserted L1-encoding genes were verified by DNA sequencing. The generated plasmids were transformed into yeast *Saccharomyces cerevisiae* strain AH22-214 (*a, leu2 his4*).

### 2.2. Expression of HPV-16-Derived L1 Proteins in Yeast

Yeast transformants harboring plasmids with genes encoding HPV-16 L1-Sc, L1-Pl, L1-Ec, L1-Hm, and L1-Pv proteins were grown in 15 mL YEPD medium (yeast extract 1%, peptone 2%, and glucose 2%) supplemented with 5 mM formaldehyde overnight at 30°C. The synthesis of recombinant proteins was induced after transferring yeast cells into induction medium 20 mL YEPG (yeast extract 1%, peptone 2%, and galactose 3%) supplemented with 5 mM formaldehyde and culturing for additional 18 h. Yeast biomass harboring recombinant proteins was harvested by centrifugation and stored at −20°C before use. Ten transformants from every group were analyzed.

### 2.3. RNA Extraction and Northern Blot Analysis

Total yeast RNA was isolated 4 hours after induction by the method described earlier [18]. The Northern blot analysis was performed by the separation of 15 *μ*g of total RNA on a 1% agarose gel containing 2.2 M formaldehyde, followed by transfer to a Hybond-N+ filter (Amersham Biosciences, Little Halfont, England) and hybridization. The DNA templates of all five L1-encoding ORFs and PGK1 ORF used for probes were generated by PCR amplification and labeled with [*α*-33P]-dATP (Hartman Analytic, Braunschrveig, Germany) by random priming of the DNA probe using a DecaLabel DNA Labeling Kit (Thermo Scientific Fermentas) according to the manufacturer's instructions. The blots were washed extensively at 65°C and L1 and PGK1 transcripts were identified by phosphorimaging.

### 2.4. Preparation of Yeast Lysates SDS-PAGE and Western Blot Analysis

10–20 mg of yeast cell pellets was resuspended in 10 volumes (vol/wt) of DB150 buffer (150 mM NaCl, 1 mM CaCl_2_, 0.001% Trition X-100 in 10 mM Tris/HCl-buffer, pH 7.2) and 1 mM PMSF. An equal volume of glass beads was added and the cells were lysed by vortexing at high speed, 8 times for 30 sec, with cooling on ice for 30 sec between each vortexing. Then an equal volume of 2 × SDS-PAGE sample buffer (125 mM Tris-HCl, pH6.8, 20% glycerol, 8% SDS, 150 mM DTT, 0.01% bromophenol blue) was added directly to the same tube, mixed and boiled immediately at 100°C for 10 minutes. 4–10 *μ*L of the prepared whole cell lysate was loaded onto SDS-polyacrylamide gel (up to 20 *μ*g protein in each lane) and sodium dodecylsulfate polyacrylamide gel electrophoresis (SDS-PAGE) was run in SDS-Tris-glycine buffer. Western blot analyses were performed according to methods described previously [19]. As a primary antibody for the immunodetection of HPV-16 L1 protein, mouse polyclonal antibody generated in-house (dilution 1 : 1000) was used. As a secondary antibody, goat antimouse IgG antibody conjugated to horseradish peroxidase diluted 1 : 3000 (Bio-Rad, Hercules, CA, USA) was used. The gels were scanned and 1 or 2 proteins bands (~50 kDa and 26 kDa) were used to determine the ratio of these proteins in the lines for evaluation of loaded yeast lysates quantitative differences. The quantitative evaluation of protein band was performed using the ImageQuant TL 1D gel analysis software (GE Healthcare).

## 3. Results and Discussion

### 3.1. Description of Codon-Optimized HPV-16 L1 Genes

The impact of synonymous codon bias on heterologous production of HPV-16 L1 protein in yeast cells was studied by expressing five L1-encoding ORFs composed of different codons. All five ORFs encoding L1 protein of HPV-16 isolate 114/K [15] were placed under the control of galactose inducible promoter using the same insertion site in the vector pFX7 [16]. The pFX7 vectors with inserted different L1-encoding ORFs were transformed into yeast strain AH22-214 and the expressions of L1 proteins were analyzed by both Northern blot and Western blot.

The codons in the authentic L1 ORF of HPV-16 isolate 114/K (L1-Pv) were optimized for either *S. cerevisiae* (L1-Sc), plant (*Solanum tuberosum; *L1-Pl), and *Homo sapiens* cells (L1-Hm) or adapted for expression in *E.coli* (L1-Ec) allowing some deviations from the strict usage of high-frequency codons to reflect the overall codon usage in the target genome. Few deviations from the strict usage of optimized codons were made also to allow the insertion or removal of recognition sites for restriction endonucleases. The majority of codons in used OFRs were modified as compared to the native HPV-16 L1 gene (L1-Pv): in L1-Sc ORF, 46.3%; in L1-Pl ORF, 51.1%; in L1-Ec ORF, 60.2%; and in L1-Hm ORF, 78.6% codons were modified while the encoded protein sequence remained unchanged (Table 1). All upstream and downstream noncoding sequences were removed in all the constructs analyzed in this study. In addition to the adjustment of codon composition, the introduced changes were likely to affect all known and unknown negative regulatory elements present in the authentic HPV-16 L1-Pv ORF.

### 3.2. Analysis of HPV-16 L1 Expression in Yeast

**Figure 1:**
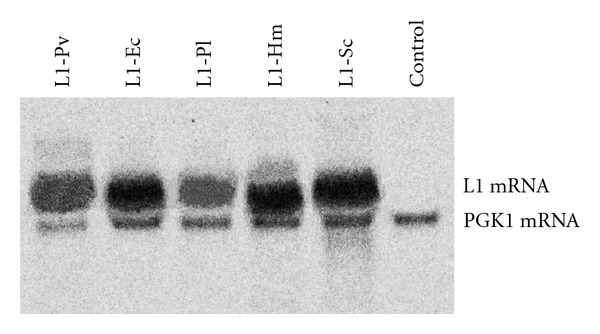
Analysis of HPV-16 L1 mRNA expression in transformed yeast cells by Northern blot. Total yeast RNA was isolated 4 h after induction from yeast transformants expressing five different HPV-16 L1 ORFs and control yeast cells transformed with the empty pFX7 vector. Fifteen micrograms of total RNA was loaded per lane and hybridized with L1-Pv, L1-Ec, L1-Pl, L1-Hm, and PGK1-control cDNA probes labeled with [*α*-33P]-dATP by random priming. The data from one representative experiment are shown. The 4 independent experiments with other randomly picked transformants in every group were performed with similar results.

To determine whether the alterations of the primary sequence of the L1 mRNAs introduced by the codon changes also affected the state and the level of transcripts, total yeast RNA was isolated from yeast cells 4 h after induction and the L1 mRNA transcription was analyzed by Northern blot. The transcripts of all five HPV-16 L1 ORFs were detected in the respective transformed yeast cells (Figure 1). Despite slightly weaker signal of L1-Pl and L1-Pv transcripts detected in Northern blot analysis, the overall mRNA produced from all five used ORFs was highly abundant and did not show apparent degradation products (Figure 1). This confirmed that codon modifications have not affected significantly the levels of transcription and the stability of HPV-16 L1 mRNA in the used yeast expression system. In contrast, the expression of L1-Pv, L1-Pl and L1-Hm in plants [13] and mammalian cells [12] was encountered with the instability of mRNA: the L1-Pv transcript was not detectable in Northern blot and most of the respective L1-Pl and L1-Hm mRNA was found to be degraded. The stability of L1-Pl and L1-Hm transcripts in plant was improved only by adding the translational enhancer 5′-leader sequence of tobacco mosaic virus [13].

**Figure 2:**
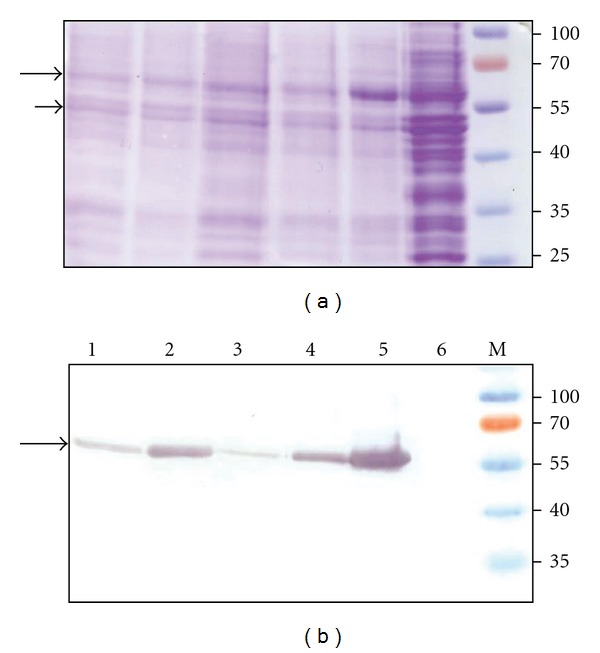
Analysis of HPV-16 L1 expression in yeast by SDS-PAGE (a) and Western blot with mouse polyclonal antibody against HPV-16 L1 protein (b). The same samples were run on each gel. In lanes: (1) crude lysate of yeast expressing ORF L1-Pv; (2) crude lysate of yeast expressing ORF L1-Ec; (3) crude lysate of yeast expressing ORF L1-Pl; (4) crude lysate of yeast expressing ORF L1-Hm; (5) crude lysate of yeast expressing ORF L1-Sc; (6) negative control sample from crude lysate of *S. cerevisiae* cells transformed with the empty vector pFX7; and M: prestained protein weight marker (Thermo Scientific Fermentas). Long arrow points to the band with HPV16 L1 protein. The protein band (~50 kDa) pointed with short arrow was used to determine the ratio of this protein in the lines for evaluation of loaded yeast lysates quantitative differences using the ImageQuant TL 1D gel analysis software (GE Healthcare). The ratio of ~50 kDa protein band in lines was 1.77, 1.00, 1.69, 1.74, 1.30, and 4.44 accordingly. The data from one representative experiment are shown. The expression level of L1 proteins in 10 randomly picked transformants in every group was alike.

In the next step, the production of heterologous HPV-16 L1 protein in transformed yeast was analyzed by both SDS-PAGE and Western blot (Figure 2). The HPV-16 L1 protein was expressed by all constructs encoding five different ORFs as demonstrated by the immunoreactivity of the respective protein bands in Western blot using HPV-16 L1-protein specific antibodies (Figure 2(b)). Western blot analysis of yeast cell lysates revealed different production levels of HPV-16 L1 protein: the highest production of L1 protein was observed in the lysate of transformed yeast expressing ORF L1-Sc (Figure 2(b), lane 5) and the moderate L1 production was detected in yeast expressing ORF L1-Ec and ORF L1-Hm (Figure 2(b), lanes 2 and 4, resp.). The diversity of HPV-16 L1 expression levels detected in the Western blot analysis but not in Northern blot suggested that the main differences in L1 protein expression levels may be addressed to the translation efficiency, rather than to gene transcription changes.

The optimization of codons for the yeast cells in the L1-Sc construct clearly had the favorable effect on L1 production. The expression of L1 protein encoded by the construct L1-Sc, carrying *S. cerevisiae* optimized codons, was the most successful with clearly detectable protein band in both SDS-PAGE and Western blot (Figures 2(a) and 2(b), lane 5). In contrast, the expression of L1-Pl construct has proven to be the most inefficient as only a faint signal of L1 protein was detected in the Western blot (Figure 2(b), lane 3). Surprisingly, the sequence of L1-Pl was the most homologous to the L1-Sc sequence because only 5 amino acids were coded by different codons in this construct. Thus, despite these small differences between the sequences encoding ORFs L1-PI and L1-Sc, there were significant differences in the production levels of L1 protein. The levels of generated HPV-16 L1 protein using other three constructs were higher than that of L1-Pl but lower than L1-Sc and could be lined from L1-Pv to L1-Hm and L1-Ec by the increasing intensity of the signal in the Western blot (Figure 2(b), lines 1, 4, and 2, resp.).

The plant and *S.cerevisiae*-optimized L1 ORFs (L1-PI and L1-Sc) sequences were comprised of similar low GC content (GC content of 35,9% and 33.0%, resp.) even lower than that found in authentic HPV-16 L1-Pv ORF (GC content of 38.7%). Meanwhile, both human- and *E. coli-*adapted L1 ORFs (L1-Hm and L1-Ec) had significantly higher GC content (65.1% and 54.5% GC, resp.). Thus, it was assumed that the efficiency of translation was not influenced by the differences in the GC content of mRNA. Most likely, the effective translation of studied HPV-16 L1 mRNAs was related to the codon usage and the availability of tRNA pool recognizing synonymous codons rather than the mRNA primary structure. Moreover, the usage of GGA (Gly), CUU (Leu) and TCA (Ser) codons as a single opportunity distinguished the L1-Pl construct from the most efficient L1-Sc and other constructs (Table 1). In the L1-Pv construct 8/35 GGA (Gly), 3/43 CUU (Leu) and 7/33 TCA (Ser) codons were also used but only in parallel with other synonymous codons and so it may loosen the pressure that was obvious for the expression of L1-Pl construct (Table 1). Although the expression level of L1-Pv protein was low, it was higher than expression of the L1-Pl despite the usage of numerous other nonfavorable codons. It is not clear which one or all three codons had this translation limiting effect because according to the frequency analysis of codons determined for overall yeast proteins (http://www.kazusa.or.jp/codon/) these codons GGA (Gly), CUU (Leu), and TCA (Ser) are moderately used. However, in highly expressed yeast genes CUU (Leu) codons are not used but few GGA (Gly) and TCA (Ser) could be found [20, 21]. Previous studies have shown that the pools of available charged tRNR may change under starvation conditions in *E. coli* [7, 8]. The results obtained in our study did not exclude the possibility that the availability of charged tRNR in yeast was also changed because of amino acid starvation caused by the expression of heterologous protein. On the other hand, the results of our study suggested that the pools of the most favorable codons in yeast were not affected and did not limit the expression level of the L1-Sc construct. Our results also supposed that despite the high toleration for adaptation of various codons used throughout the length of the sequence of heterologously expressed genes in transformed yeast, there was a significant positive correlation between the gene's expression level and the degree of its codon bias towards the favorable codon usage.

In conclusion, the HPV-16 L1 protein expression in yeast can be optimized by adjusting codon composition towards an efficient codon usage and this effect most probably is dependent on the improved translational elongation.

## Figures and Tables

**Table 1:** Codon usage in HPV-16 L1-Sc, L1-Pl, L1-Ec, Li, L1-Hm, and L1-Pv ORFs.

Amino acids	Codons	Number of the indicated codons in the ORF				
		L1-Pv	L1-Ec	L1-Pl	L1-Hm	L1-Sc
Ala	GCU	10	20	29		30
	GCC	6	4		30	
	GCA	14		1		
	GCG		6			

Arg	AGA	4	1			19
	AGG	4		19	19	
	CGU	2	13			
	CGC	4	2			
	CGA	4	1			
	CGG	1	2			

Asn	AAU	21		28		27
	AAC	7	28		28	1

Asp	GAU	18		27		27
	GAC	9	27		27	

Cys	UGU	9	1	12		12
	UGC	3	11		12	

Gln	CAA	11	19	19		19
	CAG	8			19	

Glu	GAA	14	20	19		20
	GAG	6		1	20	

Gly	GGU	15	32			35
	GGC	9	2		35	
	GGA	8	1	35		
	GGG	3				

His	CAU	8		10		10
	CAC	2	10		10	

Ile	AUU	12		21		22
	AUC		21	1	22	
	AUA	10	1			

Lys	AAA	27	32	34		34
	AAG	7	2		34	

Leu	UUA	23	1			
	UUG	5	1			43
	CUU	3		43		
	CUC					
	CUA	7	8			
	CUG	5	33		43	

Met	AUG	10	10	10	10	10

Phe	UUU	23	1	24		24
	UUC	1	23		24	

Pro	CCU	17				
	CCC	5			37	
	CCA	15	3	37		37
	CCG		34			

Ser	AGU	7	1			
	AGC	2	2		33	
	UCU	13	1			31
	UCC	4	23			
	UCA	7	3	33		2
	UCG		3			

Thr	ACU	14	2	41		41
	ACC	7	34		41	
	ACA	19	3			
	ACG	1	2			

Trp	UGG	7	7	7	7	7

Tyr	UAT	15				22
	UAC	7	22	22	22	

Val	GUU	17	30	32		32
	GUC	2	1			
	GUA	10				
	GUG	3	1		32	
